# Adolescent Girls' Agency in an Integrated Sexual and Reproductive Health and Economic Empowerment Intervention Pilot

**DOI:** 10.1002/jad.70015

**Published:** 2025-07-08

**Authors:** Meghan Cutherell, Roselyn Odeh, Seyoum Atlie, Jenna Grzeslo, Mary Phillips, Olusesan Ayodeji Makinde, Kehinde Atoloye, Andenet Haile, Albert Tele, Joy Ede, Simileoluwa Ashimolowo, Aderaw Anteneh, Claire W. Rothschild, Fifi Ogbondeminu, Abednego Musau

**Affiliations:** ^1^ Population Services International (PSI) Washington, DC USA; ^2^ Society for Family Health (SFH) Abuja Nigeria; ^3^ Population Services International (PSI) Addis Ababa Ethiopia; ^4^ BRAC International New York, NY USA; ^5^ Viable Knowledge Masters Abuja Nigeria; ^6^ Deep Dive Research Consulting Addis Ababa Ethiopia; ^7^ Independent Consultant Nairobi Kenya

**Keywords:** agency, economic empowerment, integration, power, sexual and reproductive health

## Abstract

**Introduction:**

Adolescence is a time of unique vulnerability for many girls, however, when supported to strengthen their capabilities, resources, and agency, adolescents can thrive even in adverse situations. This study sought to evaluate agency outcomes (decision‐making power, mobility, and self‐efficacy) among girls aged 15–19 participating in integrated sexual and reproductive health and economic empowerment interventions in Ethiopia and Nigeria.

**Methods:**

The study was a quasi‐experimental prospective cohort design involving two questionnaires at baseline and endline, 9 months apart, administered to intervention and comparison groups. Program effect was assessed using a difference‐in‐differences approach, modeling using linear generalized estimating equations accounting for repeated observations at the individual level and adjusted for age, marital status, education, and parity.

**Results:**

The evaluation demonstrated significant positive program effects on one agency outcome each among married girls in Ethiopia (self‐efficacy) and northern Nigeria (decision‐making power). In southern Nigeria significant positive program effects were demonstrated among unmarried girls across all agency outcomes: decision‐making power, mobility, and self‐efficacy.

**Conclusions:**

The evaluation showed promising increases in agency for girls who participated in this integrated intervention. The positive program effects seen during this relatively short pilot suggest that agency‐related gains can be made even in programs with limited implementation time.

## 1 Introduction

### 1.1 Defining Empowerment and Agency

Empowerment can be defined as ‘the expansion in people's ability to make strategic life choices in a context where this ability was previously denied to them' (Kabeer 1999). Empowerment involves a dynamic interaction between resources, agency, and achievements. *Agency* reflects the ability to define and act upon one's goals (Belachew et al. 2021; Kabeer 1999; Revollo and Portela 2019). *Resources* are the human, material, and social conditions that may expand the opportunities available to exercise choice (Kabeer 1999; Yount et al. 2016). Finally, *achievements* reflect the possible outcomes of exercising agency – such as a later age at first marriage or initiation of income generating activity (Kabeer 1999). Thus, empowerment results from individuals gaining agency and resources to pursue goals and achieve outcomes that matter to them. Without agency there is no empowerment. Individuals must be active participants in the process of change, able to formulate purposeful choices, control resources, and exercise decision‐making power (Kabeer 1999; Revollo and Portela 2019).

### 1.2 Agency in Adolescence

Transitions through adolescence pose challenges for adolescent girls, especially in contexts where deeply embedded gender norms limit girls' decision‐making power and opportunities (Banati et al. 2021; Harper et al. 2018). As girls enter adolescence, gendered norms often become rigidly enforced, leading to greater restrictions on mobility, higher burden of household care, and withdrawal from public life (Harper et al. 2018; Kågesten et al. 2016; Ricker and Ashmore 2020). Yet adolescence is also a period of great potential. Developmental changes during this period offer new opportunities for girls when they are provided with the right support (Vidyarthi et al. 2021). As investment in adolescent development has increased, there is enhanced focus on supporting adolescents to be active agents in their own development, with empowerment and agency as crucial aspects of overall wellbeing (Ross et al. 2020; Vijayaraghavan et al. 2022). Attention to positive youth development prioritizes strategies that build on adolescents' individual strengths and center adolescent voices (Banati et al. 2021; Shek et al. 2019; Vijayaraghavan et al. 2022). These approaches recognize when provided with timely and vital support adolescents can thrive, even in adverse situations, and drive change and innovation for their own health and wellbeing (Lee et al. 2012; Vijayaraghavan et al. 2022).

Though the narrowing of opportunities for adolescent girls is a trend broadly consistent across low‐ and middle‐income countries, there is still diversity in experience within different contexts. In Ethiopia, evidence demonstrates that economic growth, improved access to services, and urbanization have increased adolescents' standard of living (Young Lives & Unicef 2020). Yet, gender has a significant impact on adolescent trajectories, with girls facing increased restrictions, for example reporting needing permission to leave the house and conduct other routine activities (Jones et al. 2016, 2017). Opportunities are particularly limited when combined with other disadvantages, such as poverty and rural location (Young Lives & Unicef 2020). There are also diverse experiences within countries. In Nigeria the prevalence of early marriage, often resulting in severe restriction of girls' opportunities and mobility, is significantly higher in northern states (with some states showing upwards of 70% of girls married before age 18) than in southern states (Unicef 2020). Adolescent girls in these northern Nigerian states, especially after marriage, experience restrictions in freedom and decision‐making power, largely driven by concerns around girls' safety as well as religious and cultural norms (UNICEF 2023).

Demonstrated approaches to building agency during adolescence tend to intervene at multiple levels of the socio‐ecological framework (individual, family, community, structural) and engage stakeholders through awareness and confidence‐building activities (Vijayaraghavan et al. 2022). Successful interventions to strengthen girls' agency include: (1) development of core capabilities and soft skills such as decision‐making, negotiation, and goal setting (Chowa et al. 2021; Shek et al. 2019); (2) critical consciousness building—including awareness of rights and the role of gender in shaping opportunities and choices (Gonzalez et al. 2020; Plan International 2024; Vidyarthi et al. 2021); (3) household and community‐level dialogs with girls' gatekeepers seeking to challenge harmful social norms and unequal power relations (Ricardo et al. 2010); and (4) youth‐ and adolescent‐led defining and designing of solutions (UNICEF 2024). Often, a combination of these strategies is used together. Evidence‐based approaches to expand girls' agency tend to focus on a singular outcome area—such as sexual and reproductive health—rather than targeting agency as a holistic outcome. For example, several studies have demonstrated how improvements in agency can increase rates of contraceptive use among adolescent populations (Lassi et al. 2024; Vijayaraghavan et al. 2022).

### 1.3 Agency Dimensions and Measurement

Agency—and empowerment more broadly—is complex to measure. Measurement frameworks are biased towards use with older women and in higher income contexts (Vidyarthi et al. 2021; Zimmerman et al. 2019). Agency can be categorized along multiple dimensions (Yount et al. 2024). These include internally focused aspects of agency—for example a critical awareness of one's rights or capabilities or motivation to pursue one's aspirations (Bandura 1989; Miedema et al. 2018; Ryan and Deci 2000; Yount et al. 2024, 2016). Conversely, externally focused aspects of agency involve the establishment of solidarity, support, and unity with peers and other groups (Miedema et al. 2018). Agency measurement frameworks often combine multiple sub‐scales to assess different dimensions of agency. Most commonly sub‐scales measure aspects of self‐efficacy, decision‐making power, voice, and freedom of movement or mobility (Donald et al. 2017; Karp et al. 2020; McCarthy et al. 2022; Zimmerman et al. 2019).

### 1.4 Summary

We used a prospective cohort design with an intervention and concurrent comparison group to evaluate the effectiveness of integrated sexual and reproductive health and economic empowerment interventions in improving three dimensions of agency—decision‐making power, mobility, and self‐efficacy—among adolescent girls aged 15–19.

## 2 Materials and Methods

### 2.1 Intervention Description

The Adolescents 360 (A360) project aims to increase demand for, and voluntary use of modern contraception among adolescent girls aged 15–19. In 2021 A360 designed economic empowerment components to layer onto these existing sexual and reproductive health models (Cutherell et al. 2023). Between 2022 and 2023 these components were evaluated to assess their effect on economic and agency outcomes. This paper presents data on program effects related to girls' agency.

The specific pathways to achieve these outcomes differed by geography (Supporting information Table 1 presents the core integrated intervention elements across all three geographies and Supporting Information Tables 2 and 3 present TIDIER tables for the integrated interventions). Each pathway began with the original sexual and reproductive health intervention and girls participating in the integrated intervention were then offered the chance to continue to participate in the economic empowerment component.

In Ethiopia, married adolescent girls were invited to participate in two structured sessions—one with their husband and one individually—to identify their short and long‐term goals, emphasizing the value of young women as economic actors. Girls in the same kebele (village) then formed modified Village Savings and Loan Associations (Ksoll et al. 2016) and met weekly under the guidance of a trained mentor. After 4 weeks, girls were eligible to take out a loan from the pooled savings to fund a microenterprise. Each week, in addition to the savings and loan activities, the facilitators used participatory approaches to teach soft‐skills and business skills using a curriculum adapted from BRAC's Empowerment and Livelihoods for Adolescents (ELA) (Bandiera et al. 2012, 2025). After 16 weeks groups were eligible for a matching grant of 150% of their total savings to increase the group's loan pool if they met minimum standards for attendance, savings, and internal bookkeeping. After approximately 7 months, the facilitator phased out and associations were fully self‐governed. Around this time participants were eligible for an asset transfer to support their chosen income generation activity.

In southern and northern Nigeria, participating girls attended five upskilling group sessions (90 min each) facilitated by program mentors. The first three sessions (called the ‘primary package’) focused on supporting girls to set goals for the future and build soft skills. In the last two sessions (called the “secondary package”) girls learned business skills, including how to manage their money. Then, girls elected to learn up to two vocational skills (over 4 to 5 weeks) through an apprenticeship (southern Nigeria) or vocational training center (northern Nigeria). Girls chose a variety of trades including catering, hairdressing, shoemaking, and photography. Concurrently, mentors offered support in developing and executing a business plan. The program culminated in a large, public graduation that doubled as a marketplace for adolescent girls to display products and services.

The sexual and reproductive health interventions in Nigeria also included light‐touch program components intended to facilitate improvements in adolescent girls' soft skills. Girls mobilized for the sexual and reproductive health intervention in northern Nigeria could participate in a series of four 90‐min sessions covering topics related to health, goal setting, soft skills, and budgeting and saving. In southern Nigeria girls in the sexual and reproductive health only intervention had the choice of participating in one 90‐min session covering goal setting and a vocational skill demonstration.

### 2.2 Study Design

We employed a quasi‐experimental design consisting of an intervention and a concurrent comparison group. For the intervention group, participants received the combined sexual and reproductive health and economic empowerment intervention. A similarly sized cohort of adolescent girls of the same age group who only received a sexual and reproductive health intervention constituted the comparison group. Data was collected concurrently in both groups before participants were involved in the intervention (baseline) and 9 months after (endline) for the same participants.

### 2.3 Study Setting

Our study was based in primary health centers in Nigeria and in health posts in Ethiopia. PHCs and health posts are the lowest tier of health facilities in both countries and provide primary care services through Health Extension Workers in Ethiopia and nurses or community health workers in Nigeria. In Ethiopia, the study was based in 32 (16 intervention and 16 comparison) health posts which were randomly selected from across 16 woredas in Sidama, SNNP, and Oromia regions. In northern Nigeria, the study was conducted in Kaduna state in four intervention and three comparison primary health centers from three local government authorities—Zaria, Sabon Gari, and Igabi. In southern Nigeria, three intervention and two comparison primary health centers from Ado Odo Ota and Abeokuta South local government authorities in Ogun state were included.

### 2.4 Participant Recruitment

Participants were adolescent girls aged 15–19 residing in the catchment of selected study sites. Participants in northern Nigeria and Ethiopia were exclusively married or living with a partner as if married. In southern Nigeria, both married and unmarried girls were recruited provided they met eligibility criteria. During recruitment, girls were identified from the intervention and comparison sites by trained female mobilizers—female mentors (northern Nigeria), community mobilizers (southern Nigeria), and members of the Women Development Army or Health Extension Workers (Ethiopia). Mobilizers approached and screened girls individually for eligibility using standardized questions in a recruitment script that had been translated into the local languages. This recruitment took place in participants' homes, community or public spaces, or at the health facility. The recruitment approach was the same across intervention and comparison sites. Intervention and comparison sites were pre‐selected, with this selection considering comparability based on key factors such as socioeconomic status, rural/urban location, and insecurity. Eligible girls who expressed interest to participate in the study provided their contact details or physical addresses which were recorded in a confidential recruitment sheet and relayed to a trained female data collector. Female data collectors contacted the individuals and scheduled an in‐person meeting at the study site. During the in‐person contact, potential participants were informed about the study objectives, time commitment, confidentiality, and study risks and benefits before they provided signed informed consent or assent. Adolescent girls 18–19 years and emancipated minors (married adolescents) provided consent. A waiver of parental consent was granted during ethical approval to minimize potential privacy risks to participants given the inclusion of sexual and reproductive health topics in the evaluation and unmarried minors provided assent in lieu of consent (applicable for southern Nigeria only). Before baseline, participants provided consent for their participation both in the baseline and endline surveys. No re‐consenting was conducted at endline.

In both groups, data collection was conducted before the participants' involvement in the interventions. After potential study participants were contacted and screened by female data collectors and the baseline survey was administered, these participants were recontacted by the female mobilizers and provided the opportunity to participate in the intervention. As an intent‐to‐treat analysis, girls who chose to engage in the study but not in the intervention were retained in the group to which they were classified at the beginning of the study regardless of their exposure.

### 2.5 Sample Size and Attrition

The sample comprised 1457 girls for the intervention group (Ethiopia: 400, Ogun: 501, and Kaduna: 556) and 1319 girls for the comparison group (Ethiopia: 400, Ogun: 426, and Kaduna, 493). At endline 28.3% of the sample in southern Nigeria and 19.8% of the sample in northern Nigeria were lost to follow‐up. No girls were lost to follow‐up in the Ethiopia sample—this was attributed to the close knit small rural communities and minimal movement of married girls. Across Ogun and Kaduna there was greater loss to follow up in the comparison group than in the intervention group. Several significant differences between those followed up with and those lost to follow up were identified through a differential attrition analysis including age, baseline economic behaviors, and baseline agency‐related scores (supporting information Tables 4 and 5). In addition, we conducted an analysis with propensity score weights, generated out of a logistic model with the state of follow‐up as the outcome and using the covariates used for the difference‐in‐difference (DiD) models. These weights were then applied to the DiD model using generalized estimating equations (GEE). The results of this analysis were robust and are included in supporting information Table 6.

### 2.6 Data Collection Procedures

A structured quantitative questionnaire was fielded to participants at baseline. The questions in the baseline survey were repeated at endline, which was conducted 9 months after baseline in all three geographies, excluding introductory socio‐demographic questions. At endline participants in both groups were also asked about their exposure to the different intervention elements (intervention group) or similar interventions (comparison group). Trained female data collectors and supervisors were deployed to manage the field data collection. Data was collected using the computer assisted personal interview (CAPI) approach. Baseline and endline questionnaires were pre‐coded into electronic forms using Open Data Kit (ODK). All questionnaires were translated from English into local languages (Amharic and Afan Oromo in Ethiopia, Hausa and Yoruba in Nigeria) and back‐translated to ensure accuracy. Survey questions were pre‐tested with program participants in non‐survey areas to screen for survey programming errors and verify competency of data collectors. Questionnaires were administered in‐person except for 51 participants in southern Nigeria at endline which were conducted over the phone because participants had moved out of the study catchment areas and were unreachable in person. Survey interviews at baseline and endline were conducted within spaces conferring auditory and visual privacy. Surveys took approximately 30–40 min at both time points. In each round, participants were provided with a travel reimbursement equivalent to $2 in the local currencies upon the completion of the survey.

### 2.7 Measures

The questionnaire collected participants' socio‐demographic characteristics and specific measures for agency outcomes. Socio‐demographic characteristics collected were age, marital status, education level, and number of children birthed. Three scales were employed as measures of agency (Kabeer 1999).

1. **Decision‐making power:** We adopted a measure from Austrian and Ghati (2010), to assess decision‐making power. Participants were given a set of four or six statements regarding important life decisions and asked the question “*who mostly makes decisions about the following…?*” before each statement. All participants were asked about *socializing outside the home*, *going to school or studying*, *deciding whether one should have sex*, and *engaging in an income‐generating activity*. Participants who were unmarried were also asked who decides *who they will marry* and *when they will get married*. The answer options for each statement were: 1 = *I do*, 2 = *I do together with someone else*, or 3 = *Someone else does*.
2. **Mobility:** We adopted the likert‐type scale from Austrian and Ghati (2010), to assess the frequency with which participants required permission to perform certain actions. Participants were given a set of four statements related to aspects of mobility (leaving the house, visiting a friend, looking for work and spending money) and asked to indicate how often they required permission to perform these actions. Participants responded with answer options 1 = *Never*, 2 = *Rarel*y, 3 = *Sometimes*, or 4 = *Always*.
3. **Self‐efficacy**: We used the General Self‐Efficacy Scale (Schwarzer & Jerusalem 1995), a 10‐item global measure of self‐efficacy that assesses optimistic self‐belief in one's ability to cope with difficult tasks, demands, or adversity and achieve objectives. Respondents rated each statement on a 4‐point likert scale: 1 = *not at all true*, 2 = *hardly true*, 3 = *moderately true*, and 4 = *exactly true*.

### 2.8 Data Analysis

Descriptive analysis was used to generate summaries for baseline socio‐demographic characteristics. We tested for baseline differences between intervention and comparison groups using Pearson's Chi‐square test. For the measures of agency, scores for decision‐making power and mobility were reverse coded so that a higher score denoted higher agency (independent decision making and a lesser need for permission to perform certain tasks, respectively). The self‐efficacy scoring structure was retained with a high score denoting high self‐confidence. Average scores for each individual were generated and ranged from 1 to 3 for decision‐making power and 1 to 4 each for self‐efficacy and mobility.

Decision‐making power and mobility were analyzed both as continuous and as categorical measures. Self‐efficacy was analyzed only as a continuous measure. We conducted DID analyzes for each scale using GEE models, which include the average scores, a dummy variable indicating time period, a dummy variable for group (intervention vs. comparison), and the DID estimator, modeled as the interaction between time period and group. The GEE DID estimator represents the population‐level average difference in agency between baseline and endline in the intervention versus comparison group. Adjusted DID models included a priori selected control variables based on literature (age, education level, number of children, and marital status–southern Nigeria only). These variables were all categorical. Age and number of children were defined as a numeric response across geographies. Responses regarding highest level of education completed were non‐numeric, recorded as none, primary, secondary, or above secondary. The GEE models were specified as linear models with panel fixed effects with robust standard errors. We estimated unadjusted and adjusted beta coefficients with corresponding 95% confidence intervals.

To understand agency levels of study participants and movement between levels from baseline to endline, we conducted an exploratory analysis of decision‐making and mobility as categorical variables (key depicted in supporting information Table 7). Categorizations for decision‐making power and mobility were combined to form five composite categories (supporting information Figure 1). The results from this analysis were depicted on Sankey plots showing the change in the proportion of participants in each category between baseline and the endline separately for each group and study geography.

### 2.9 Ethical Considerations

Research approval was granted by the PSI Research Ethics Board (approval No. 10.2022 for Ethiopia and 09.2022 for Nigeria). Additionally, approvals were received from a relevant local authority in each study geography: Ethiopian Public Health Institute (Ethiopia) approval No. EPHA/06/808/22, National Health Research Ethics committee (Nigeria) approval No. NHREC/01/01/2007‐06/05/2022, State Health Research Ethics Committee, Ogun State, approval No. HPRS 388/455, and Health Research Ethics committee of Kaduna State Ministry of Health, approval No. NHREC/17/03/2018.

## 3 Results

### 3.1 Sample Characteristics and Program Exposure

At endline, nearly all study participants in Ethiopia and Kaduna were married and the majority were aged 18–20 (85%) and had at least one child (86%) (Table 1). In Ogun more than 90% of participants were unmarried, half were aged 15–17, and only 30% had a child. The proportion of study participants currently in school was greatest in Ogun (61.5%), followed by Kaduna (30.3%), then finally Ethiopia (3.4%).

**Table 1:** Participant demographic characteristics and intervention exposure at endline by geography and group.

	Ethiopia			Kaduna			Ogun		
	Intervention	Comparison		Intervention	Comparison		Intervention	Comparison	
	*N* (%)	*N* (%)	*p*‐value	*N* (%)	*N* (%)	*p*‐value	*N* (%)	*N* (%)	*p*‐value
Age									
15–17	87 (21.8%)	77 (19.3%)	0.38	29 (6.1%)	59 (16.1%)	**< 0.001**	222 (54.7%)	112 (43.4%)	**0.005**
18–20	313 (78.3%)	323 (80.8%)		445 (93.9%)	308 (83.9%)		184 (45.3%)	146 (56.6%)	
School status									
In school	10 (2.5%)	17 (4.3%)	0.17	128 (27.2%)	119 (34.5%)	0.026	248 (61.4%)	159 (61.6%)	0.95
Out of school	390 (97.5%)	383 (95.8%)		342 (72.8%)	226 (65.5%)		156 (38.6%)	99 (38.4%)	
Level of education									
None	10 (2.5%)	17 (4.3%)	0.29	11 (2.3%)	91 (24.8%)	**< 0.001**	1 (0.2%)	0 (0.0%)	0.84
Primary	286 (71.5%)	272 (68.0%)		9 (1.9%)	57 (15.5%)		12 (3.0%)	7 (2.7%)	
Secondary	94 (23.5%)	105 (26.3%)		368 (77.6%)	193 (52.6%)		367 (90.6%)	237 (91.9%)	
Above secondary	10 (2.5%)	6 (1.5%)		86 (18.1%)	26 (7.1%)		25 (6.2%)	14 (5.4%)	
Marital status									
Unmarried	2 (0.5%)	10 (2.5%)	**0.020**	0 (0.0%)	0 (0.0%)	n/a	388 (95.8%)	236 (91.8%)	0.032
Married	398 (99.5%)	390 (97.5%)		474 (100.0%)	367 (100.0%)		17 (4.2%)	21 (8.2%)	
Parity									
0	43 (10.9%)	100 (25.4%)	**< 0.001**	32 (6.8%)	52 (14.2%)	**< 0.001**	308 (75.9%)	159 (61.6%)	**< 0.001**
1	248 (62.8%)	214 (54.5%)		206 (43.5%)	92 (25.1%)		31 (7.6%)	32 (12.4%)	
≥ 2	104 (26.3%)	79 (20.1%)		236 (49.8%)	223 (60.8%)		67 (16.5%)	67 (26.0%)	

*Note:* Bolded values reflect *p*‐values associated with variables that have demonstrated statistical significance.

Participants in the intervention group across all three geographies showed high exposure to the integrated intervention (Table 2). In Ethiopia, 99% or more of study participants completed at least one future mapping session and participated in a savings group. Most (71%) participants attended all savings group sessions but 19.7% missed one to two sessions, and 9.8% missed three or more sessions. In Kaduna 90.9% of study participants completed all primary package sessions, 92.2% completed all secondary package sessions, and 97.7% participated in the vocational skills sessions. In Ogun, 86.2% of participants completed all primary package sessions, 90.9% completed all secondary package sessions, and 97.8% participated in the vocational skills sessions.

**Table 2:** Exposure to economic empowerment activities in intervention and comparison groups across geographies.

	Ogun	Kaduna		Ethiopia
	*n* (%)	*n* (%)		*n* (%)
Intervention group				
Participated in life mapping/goal setting			Participated in goal setting session	
Yes	398 (98.0%)	466 (98.3%)	With husband	397 (99.5%)
No	8 (2.0%)	6 (1.3%)	By yourself/with other girls	362 (90.5%)
Refused/don't know		2 (0.4%)		
Participated in primary package sessions			Participated in savings groups	
Some sessions	53 (13.1%)	40 (8.4%)	Ever participation	396 (99.2%)
All sessions	350 (86.2%)	431 (90.9%)	Still participating currently	393 (99.0%)
Refused/don't know	3 (0.7%)	3 (0.6%)		
Participated in secondary package sessions			Number of savings group sessions missed	
Some sessions	34 (8.4%)	32 (6.8%)	None	279 (70.5%)
All sessions	369 (90.9%)	437 (92.2%)	1–2	78 (19.7%)
Refused/don't know	3 (0.7%)	1 (0.2%)	3+	39 (9.8%)
Participated in vocational skills sessions			Participated in economic empowerment sessions	
Yes	397 (97.8%)	463 (97.7%)	Some sessions	71 (17.8%)
No	9 (2.2%)	10 (2.1%)	All sessions	329 (82.3%)
Refused/don't know		1 (0.2%)		
Comparison group				
Any program to increase skills in prior 9 months			Any program to increase skills in prior 9 months	
Yes	73 (28.3%)	217 (59.1%)	Yes	73 (18.3%)

In the comparison group there was also some self‐reported exposure to economic empowerment related programming. In Ethiopia, 18.3% of comparison group participants indicated they participated in a training or skills‐related program in the last 9 months. In Ogun and Kaduna 28.3% and 59.1% of participants in the comparison groups respectively indicated participation in a training or skills program. Most (81%) of these exposed participants in the Kaduna comparison group mentioned MMA's skills sessions (part of the sexual and reproductive health only intervention) as the source of this training.

### 3.2 Agency Outcomes

**Figure 1:**
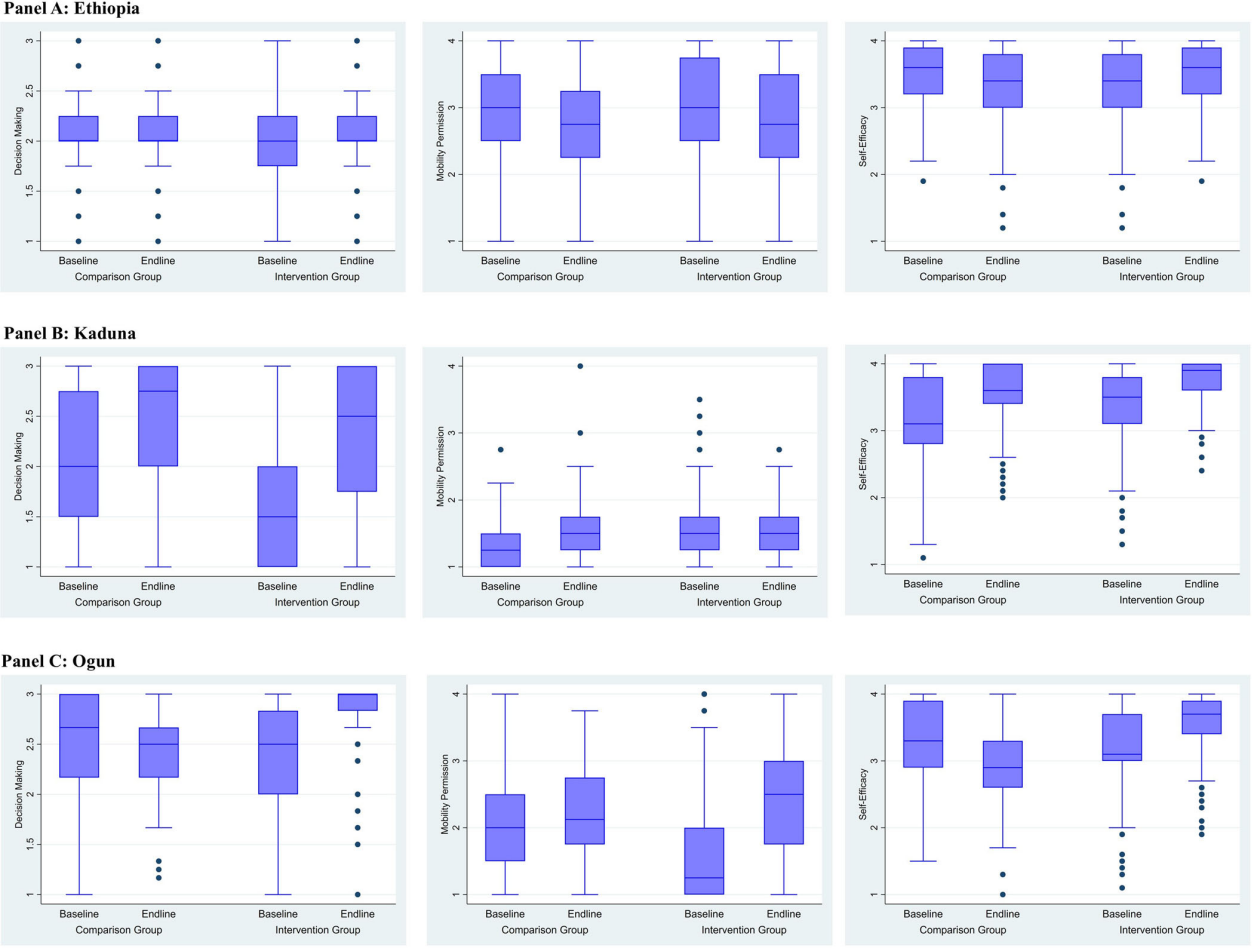
Box plots for agency related measures by group and time point.

In Ethiopia (Figure 1, Panel A), both groups saw increases in the mean score for decision‐making power (0.10 intervention and 0.02 comparison) and mobility (0.18 intervention and 0.17 comparison). The mean self‐efficacy score in the intervention group increased (0.18) while the comparison group saw a corresponding decrease (−0.18). In Kaduna (Figure 1, Panel B), both groups saw increases in mean scores across all three outcomes. Decision‐making power saw the greatest increases (0.72 intervention and 0.42 comparison), followed by self‐efficacy (0.38 intervention and 0.44 comparison), then mobility (0.07 intervention and 0.16 comparison). In Ogun (Figure 1, Panel C), the intervention group showed an increased mean score on all three outcomes ‐ decision‐making power (0.42), mobility (0.85), and self‐efficacy (0.34). The comparison group showed minimal or negative change on all outcomes—decision‐making power (−0.10), mobility (0.04), and self‐efficacy (−0.37).

The unadjusted DID analysis demonstrated significant positive program effects on self‐efficacy in Ethiopia (β = 0.36, 95% confidence interval [CI]: 0.27; 0.45), decision‐making power in Kaduna (β = 0.32, [95% CI: 0.23; 0.42]), and on all three outcomes in Ogun (β = 0.59, [95% CI: 0.50; 0.68]) decision‐making power, β = 0.90 (95% CI: 0.75; 1.05) mobility, and (β = 0.75 [95% CI: 0.64; 0.87] self‐efficacy) (Table 3, Panel A). Beta coefficients in the analysis reflect the differences in the change in the mean score between the intervention and comparison group between baseline and endline. For example, in Ethiopia the intervention group saw a change in the mean self‐efficacy score which was 0.36 points higher than the change in the control group. In Kaduna there was a negative program effect on mobility, though the magnitude of the estimate was small (β = −0.08 (95% CI: −0.14; −0.02)). All significant findings were retained in the adjusted model (Table 3, Panel B) with minor variations in the beta coefficients between the unadjusted and adjusted DID models. In the adjusted model, older age was associated with a greater increase in the mean decision‐making power and mobility scores between baseline and endline across most geographies while in Ogun, married girls demonstrated a decreased mean decision‐making power scores between the two time points. In Kaduna, higher education levels were associated with greater increases in mean scores for decision‐making power, mobility, and self‐efficacy. Higher parity was associated with larger increases in decision‐making power and mobility scores in Ogun and with decreases in mean scores on the decision‐making power scale in Kaduna.

**Table 3:** Unadjusted and adjusted difference‐in‐difference (DiD) for agency‐related outcomes in economic empowerment pilot evaluation.

Panel A: Unadjusted DiD																		
	Decision‐making power						Mobility						Self‐efficacy					
	Ethiopia		Kaduna		Ogun		Ethiopia		Kaduna		Ogun		Ethiopia		Kaduna		Ogun	
	*n* = *1600*		*n* = *1890*		*n* = *1591*		*n* = *1600*		*n* = *1890*		*n* = *1591*		*n* = *1600*		*n* = *1890*		*n* = *1591*	
	Baseline	Endline	Baseline	Endline	Baseline	Endline	Baseline	Endline	Baseline	Endline	Baseline	Endline	Baseline	Endline	Baseline	Endline	Baseline	Endline
	*M (SD)*	*M (SD)*	*M (SD)*	*M (SD)*	*M (SD)*	*M (SD)*	*M (SD)*	*M(SD)*	*M (SD)*	*M (SD)*	*M (SD)*	*M (SD)*	*M (SD)*	*M (SD)*	*M (SD)*	*M (SD)*	*M (SD)*	*M (SD)*
Intervention	2.04 (0.48)	2.15 (0.36)	1.61 (0.55)	2.33 (0.67)	2.44 (0.45)	2.86 (0.25)	2.03 (0.88)	2.21 (0.82)	1.48 (0.43)	1.55 (0.37)	1.57 (0.66)	2.41 (0.66)	3.34 (0.53)	3.52 (0.43)	3.40 (0.50)	3.78 (0.31)	3.22 (0.56)	3.56 (0.43)
Control	2.08 (0.43)	2.10 (0.37)	2.10 (0.68)	2.52 (0.59)	2.55 (0.43)	2.45 (0.40)	2.11 (0.82)	2.28 (0.78)	1.32 (0.29)	1.48 (0.36)	2.10 (0.80)	2.14(0.69)	3.52 (0.43)	3.34 (0.53)	3.16 (0.63)	3.59 (0.38)	3.31 (0.55)	2.93 (0.53)
Unadjusted DiD	0.08 (−0.002; 0.16)		**0.32** *** **(0.23; 0.42)**		**0.59** *** **(0.50; 0.68)**		0.003 (−0.16; 0.17)		−**0.08** * **(−0.14; −0.02)**		**0.90** *** **(0.75; 1.05)**		**0.36** *** **(0.27; 0.45)**		−0.03 (−0.11; 0.06)		**0.75** *** **(0.64; 0.87)**	

Panel B: Adjusted DiD																		
	Decision‐making power						Mobility						Self‐efficacy					
	Ethiopia		Kaduna		Ogun		Ethiopia		Kaduna		Ogun		Ethiopia		Kaduna		Ogun	
	*n* = *1600*		*n* = *1890*		*n* = *1591*		*n* = *1600*		*n* = *1890*		*n* = *1591*		*n* = *1600*		*n* = *1890*		*n* = *1591*	
Time* Intervention (DiD)	0.07 (−0.01; 0.15)		0.34*** (0.24; 0.43)		0.61*** (0.52; 0.70)		−0.01 (−0.17; 0.15)		−0.08* (−0.14; −0.01)		0.97*** (0.82; 1.11)		0.35*** (0.25; 0.44)		−0.03 (−0.12; 0.06)		0.77*** (0.66; 0.89)	
Program exposure																		
Comparison	Ref.		Ref.		Ref.		Ref.		Ref.		Ref.		Ref.		Ref.		Ref.	
Intervention	−0.02 (−0.08; 0.05)		−0.65*** (−0.74; ‐0.56)		−0.17*** (−0.24; −0.10)		−0.05 (−0.17; 0.07)		0.12*** (0.07; 0.18)		−0.64*** (−0.76; ‐0.53)		−0.18*** (−0.25; ‐0.11)		0.10* (0.02; 0.17)		−0.14** (−0.22; −0.04)	
Period			0															
Baseline	Ref.		Ref.		Ref.		Ref.		Ref.		Ref.		Ref.		Ref.		Ref.	
Endline	0.01 (−0.05; 0.09)		0.39*** (0.32; 0.46)		−0.17*** (−0.25; −0.10)		0.13* (0.03; 0.24)		0.14*** (0.09; 0.18)		−0.09 (−0.22; 0.03)		−0.18*** (−0.24; ‐0.10)		0.41*** (0.34; 0.49)		−0.44*** (−0.54; −0.34)	
Age																		
Age 15–17	Ref.		Ref.		Ref.		Ref.		Ref.		Ref.		Ref.		Ref.		Ref.	
Age 18–19	0.18*** (0.14; 0.23)		0.13* (0.03; 0.22)		0.08** (0.04; 0.12)		0.29*** (0.20; 0.38)		0.04 (−0.01; 0.09)		0.11** (0.03; 0.19)		0.01 (−0.18; 0.21)		0.09* (0.02; 0.16)		0.02 (−0.04; 0.07)	
Married/living as married																		
No	Ref.		N/A		Ref.		Ref.		N/A		Ref.		Ref.		N/A		Ref.	
Yes	−0.24 (−0.56; 0.06)		N/A		−0.22*** (−0.32; −0.11)		−0.72** (−1.26; ‐0.18)		N/A		−0.19* (‐0.37; −0.20)		0.01 (−0.18; 0.21)		N/A		−0.03 (−0.16; 0.10)	
Highest level of education																		
No formal education	Ref.		Ref.		Ref.		Ref.		Ref.		Ref.		Ref.		Ref.		Ref.	
Primary	−0.10* (−0.18; ‐0.014)		−0.02 (−0.17; 0.13)		−0.16 (−0.46; 0.13)		−0.08 (−0.26; 0.10)		0.04 (−0.03; 0.10)		0.26 (−0.13; 0.65)		−0.01 (−0.13; 0.12)		−0.04 (−0.16; 0.07)		−0.16 (−0.72; 0.39)	
Secondary	−0.07 (−0.16; 0.02)		0.29*** (0.18; 0.41)		−0.02 (−0.27; 0.22)		−0.04 (−0.23; 0.15)		0.05* (0.001; 0.11)		0.51** (0.18; 0.84)		0.01 (−0.12; 0.14)		0.25*** (0.18; 0.34)		−0.03 (−0.16; 0.10)	
Above secondary	−0.08 (−0.25; 0.10)		0.36*** (0.22; 0.50)		0.02 (−0.23; 0.27)		0.08 (−0.29; 0.46)		0.10** (0.03; 0.17)		0.70*** (0.31; 1.09)		−0.04 (−0.27; 0.20)		0.30*** (0.21; 0.40)		0.11 (−0.42; 0.65)	
Parity																		
0	Ref.		Ref.		Ref.		Ref.		Ref.		Ref.		Ref.		Ref.		Ref.	
1	0.03 (−0.01; 0.09)		−0.16** (−0.24; ‐0.07)		0.08* (0.007; 0.15)		0.09 (−0.01; 0.19)		−0.01 (−0.07; 0.04)		0.36*** (0.21; 0.50)		0.04 (−0.02; 0.10)		−0.05 (−0.13; 0.03)		0.16** (0.06; 0.25)	
≥ 2	0.05 (−0.01; 0.09)		−0.15** (‐0.24; −0.06)		0.13*** (0.07; 0.19)		0.20** (0.08; 0.32)		0.03 (−0.03; 0.08)		0.35*** (0.24; 0.46)		0.01 (−0.06; 0.08)		−0.08 (−0.15; 0.002)		0.06 (−0.004; 0.13)	

* *p* < 0.05

** *p* < 0.01

*** *p* < 0.001.

Supporting information Table 6 presents the unadjusted and the adjusted results of the DiD analysis using propensity score weights to account for differential loss‐to‐follow‐up. These results show minimal differences compared to the analysis without propensity score weights.

**Figure 2:**
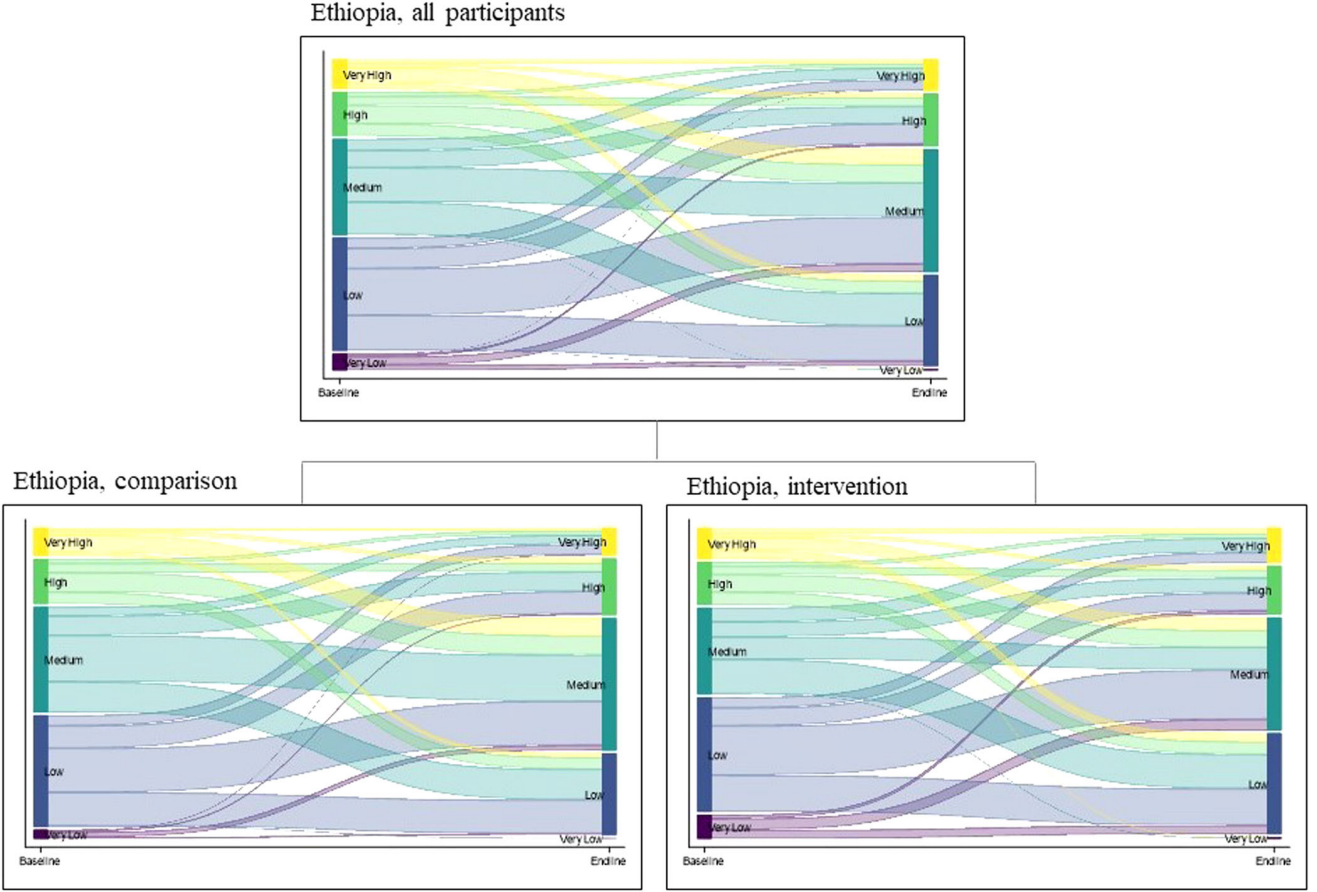
Changes in agency categories between time points in Ethiopia.

At baseline, study participants in Ethiopia were most likely to be classified as having low (38%) or medium (32%) agency (Figure 2). This was consistent across groups. Both groups saw movement from the low agency category into the medium and high agency categories between baseline and endline. The shifts were nearly equal in the comparison and intervention groups, with a 12% decrease in the low/very low categories, a 9% increase in the medium agency category, and a 3% increase in the high/very high categories. By endline, study participants were most likely to be classified as having medium agency (41%).

**Figure 3:**
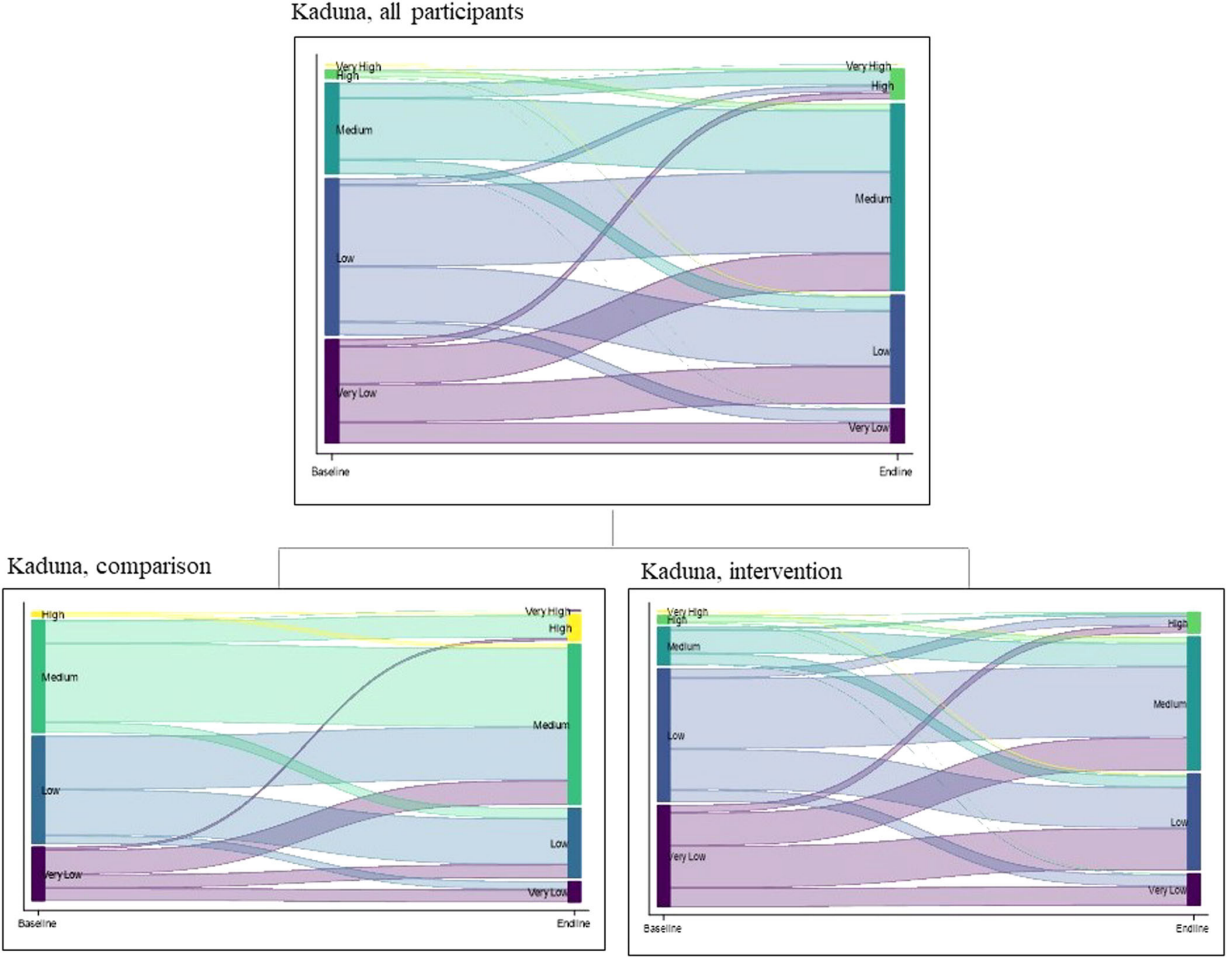
Changes in agency categories between time points in Kaduna.

In Kaduna (Figure 3) 43% of the study participants at baseline were categorized as having low agency and an additional 29% as having very low agency. The intervention group had lower baseline levels of agency, with 83% in the low or very low agency categories compared with 58% in the comparison group. Both groups saw movement from baseline to endline from the low agency to medium agency categories. By endline 57% of the study population in the comparison group and 47% of the study population in the intervention group had medium agency, demonstrating greater movement out of these low agency categories in the intervention group.

**Figure 4:**
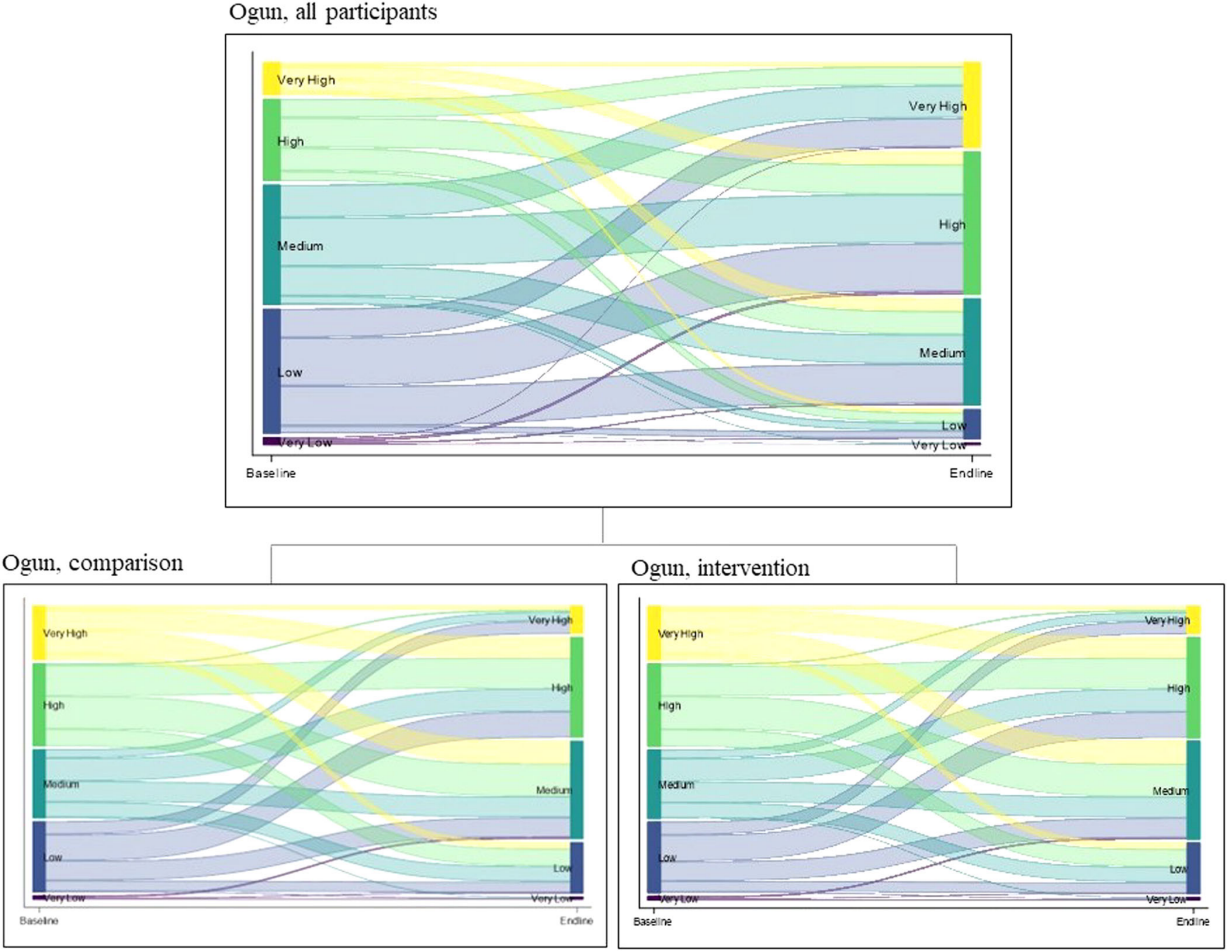
Changes in agency categorization between time points in Ogun.

In Ogun (Figure 4) study participants were most likely to be categorized as having low (34%) or medium (33%) agency, with the comparison group having higher baseline levels of agency than the intervention group. Around one quarter (27%) of participants in the comparison group were categorized as having low or very low agency at baseline compared to 42% in the intervention group. The intervention group showed a reduction in the very low/low agency categories from 42% at baseline to 2% at endline and an increase in the high agency category from 20% at baseline to 73% at endline. The comparison group showed some reductions in the low agency category from baseline (27%) to endline (19%) but most movement happened into the medium agency category, with a small decrease in the high agency category (49% at baseline to 46% at endline).

Numeric values depicted in Figure 2 through Figure 4 are found in supporting information Table 8.

## 4 Discussion

### 4.1 Evaluation Results

This evaluation demonstrated mixed results in improved agency outcomes for girls participating in the integrated intervention. In some cases, there were promising shifts, for example mean scores on all agency outcomes increased from baseline to endline across all intervention groups. However, this was not always greater than the positive change in the comparison group, leading to a lack of statistically significant program effect. There are several potential explanations for these findings. While girls in the comparison group did not receive the economic empowerment interventions, the sexual and reproductive health interventions that they did receive could have influenced agency outcomes, for example, through goal setting, joint counseling sessions with husbands, and soft skills. Increased levels of critical consciousness could also have led intervention group participants to respond more critically to prompts within the agency‐related measures (Maker Castro et al. 2022), resulting in underestimated program effectiveness. Although this paper presents the agency‐related outcomes for this program evaluation, the interventions were also designed to influence economic and sexual and reproductive health outcomes. These outcomes will be assessed in separate publications and understanding these holistic results will provide a comprehensive understanding of intervention effectiveness.

Among agency outcomes, mobility was least likely to demonstrate program effects. Considering where each of these dimensions fall within the socio‐ecological framework, this is not necessarily surprising. Self‐efficacy, at the individual level, and decision‐making power, at the household level, were areas where the project had direct influence. In Kaduna and Ogun, the integrated interventions engaged girls' key influencers to support their participation and decision‐making. In Ethiopia, the intervention included a joint goal setting session with girls and their husbands along with an individual goal setting session for each participant. Mobility has household‐level elements but is strongly influenced by community and societal norms that are more difficult for a project to influence without substantial attention (Jones et al. 2019). Most programs do not see changes in areas that are outside of their core implementation design (Díaz‐Martin et al. 2023). Therefore, more intentional programmatic components would have needed to be incorporated to influence mobility. This is something that we were unable to do within a 9‐month pilot. Programs should also give attention to the interplay between agency‐related dimensions, as lack of progress in one may limit the potential for changes to be realized in others.

While decision‐making power is typically measured assuming individual decision‐making is preferable to joint decision‐making, preferences may vary widely across contexts and individuals. For example, our insights during program design demonstrated that some married girls in Ethiopia preferred joint decision‐making over individual decision‐making. Therefore, the integrated intervention reflected activities that promoted joint decision‐making. As we did not have enough evidence to demonstrate this as a universal preference, we retained individual decision‐making as the desired outcome, also allowing us to keep the same analysis approach across all three geographies. However, we echo others' recommendations to design and evaluate programs in ways that support girls to define and work towards achieving their own stated preferences regarding decision‐making, particularly in the context of adolescent marriage, rather than prescribing one model of decision‐making as “right” for all (Tomar et al. 2021). This also aligns with emerging work around ‘preference‐aligned’ measures of program success within the sexual and reproductive health field (Rothschild et al. 2024).

Heterogeneity in program effectiveness across geographies (particularly mixed results in Ethiopia and Kaduna) may be explained by differences in the intervention design and in the target populations. In Ogun, where the intervention engaged unmarried girls, we saw the greatest intervention effects on agency outcomes, suggesting that improvements in agency among this cohort might be more easily attained. This could be due to the less restrictive normative environment in southern Nigeria compared to northern Nigeria and Ethiopia. We conclude that the interventions in Ethiopia and Kaduna needed a longer engagement with married girls and their key influencers (husbands and other community gatekeepers) and a more intentional approach to values clarification/attitude transformation around girls' right to make decisions, exercise their voice, and have freedom of mobility to have affected changes across all agency‐related outcomes. However, even limited change is meaningful in the context of an intervention designed to influence a range of holistic outcomes. Changing the underlying norms that create restrictive environments for married adolescent girls takes time and significant effort. This may not be achieved by one partner alone but could be achieved through pooled efforts from multiple partners supporting outcomes for adolescents across various domains.

### 4.2 Study Strengths and Limitations

Since intervention and comparison sites had already been selected at the time of study design, we used a quasi‐experimental, non‐randomized evaluation design. While our DiD approach accounts for time‐invariant confounding (measured and unmeasured), there is a possibility of unmeasured confounding by time‐varying factors differentially impacting the intervention and comparison groups because of the site preselection rather than randomization. Furthermore, given the collection of data at only two time points, we are unable to empirically assess whether time trends in the outcomes of interest were following parallel, pre‐intervention slopes—an assumption of the DID design. As an intent‐to‐treat analysis, the study should not have over‐estimated effects given self‐selection bias of participants into the intervention, however, we cannot rule out the possibility of differentiated self‐selection of participants into the study between the intervention and comparison sites. Additionally, as we did not ask time‐variant socio‐demographic questions (such as pregnancy or school status) at endline, these factors could have influenced the results but were not controlled for in the analysis.

The relatively short period (9 months) between baseline and endline is both a strength and limitation of the study. The duration of the pilot was shortened to respond to gaps in the evidence base suggesting more research is needed on different program dosages (Temin and Heck 2020), yet the short duration limited the ability to see long‐term program effects. The period for this evaluation is shorter than similar economic empowerment programs evaluated in the evidence base which tend to be 1 year or more in duration (Haberland et al. 2021). As the integrated interventions layered group‐based programming and individual coaching and mentorship components, we are unable to determine which elements of the interventions had the greatest influence on program outcomes. This difficulty in disentangling the impact of individual elements of multicomponent interventions is established in the evidence base and warrants further exploration (Díaz‐Martin et al. 2023; Haberland et al. 2021).

High baselines may have influenced the lack of significant effects on certain outcomes, particularly self‐efficacy where in nearly all cases the average mean score was 3 or more out of a max of 4. High loss to follow up at endline in Nigeria, particularly in Ogun, may also have influenced results. Our differential attrition analysis (Supporting information Tables 4 and 5) indicated that in Ogun and Kaduna those who were lost to follow up were more likely to be classified as having low agency at baseline. There could be additional differences between groups that we did not assess that influenced the presence or lack of significant program effect.

### 4.3 Conclusion and Recommendations

The positive program effects demonstrated suggest that agency‐related gains can be made even in programs with limited implementation time. However, particularly for married adolescent girls we recommend more focused, longitudinal research and programming to understand the unique considerations faced by this population in strengthening their agency. We reinforce the need for refining adolescent‐focused and streamlined measures of agency—greater understanding is needed regarding how agency‐related dimensions interact with and influence each other in the pathway to greater empowerment.

## Author Contributions

**Meghan Cutherell:** conceptualization, funding acquisition, methodology, project administration, resources, supervision, validation, visualization, writing – original draft preparation, writing – review and editing. **Roselyn Odeh:** conceptualization, investigation, project administration, supervision, validation, writing – review and editing. **Seyoum Atlie:** conceptualization, investigation, project administration, supervision, validation, writing – review and editing. **Jenna Grzeslo:** conceptualization, data curation, formal analysis, investigation, methodology, supervision, validation, writing – review and editing. **Mary Phillips:** conceptualization, supervision, validation, visualization, writing – original draft preparation, writing – review and editing. **Olusesan Ayodeji Makinde:** data curation, formal analysis, investigation, methodology, project administration, validation, writing – review and editing. **Kehinde Atoloye:** data curation, formal analysis, investigation, methodology, project administration, validation, writing – review and editing. **Andenet Haile:** data curation, formal analysis, investigation, methodology, project administration, validation, writing – review and editing. **Albert Tele:** data curation, formal analysis, validation, visualization, writing – original draft preparation, writing – review and editing. **Joy Ede:** conceptualization, project administration, resources, validation, writing – review and editing. **Simileoluwa Ashimolowo:** conceptualization, project administration, resources, validation, writing – review and editing. **Aderaw Anteneh:** conceptualization, methodology, project administration, supervision, validation, writing – review and editing. **Claire W. Rothschild:** formal analysis, methodology, validation, visualization, writing – original draft preparation, writing – review and editing. **Fifi Ogbondeminu:** conceptualization, funding acquisition, methodology, validation, writing – review and editing. **Abednego Musau:** conceptualization, data curation, formal analysis, methodology, project administration, resources, supervision, validation, visualization, writing – original draft preparation, writing – review and editing.

## Consent

Written informed consent or assent was obtained from all participants prior to their involvement.

## Conflicts of Interest

The authors declare no conflicts of interest.

## Supporting information

Agency in integrated pilot supplement.

Supplement Figure 1 Composite agency categories.

## Data Availability

Data sets associated with this article are publicly available through the figshare online platform under the title “A360 Economic Strengthening Pilot Evaluation Data Sets” and DOI: https://doi.org/10.6084/m9.figshare.29390573.v1.
