# Supplementary material for: Adolescent Girls' Agency in an Integrated Sexual and Reproductive Health and Economic Empowerment Intervention Pilot
Source: J Adolesc. 2025 Jul 8;97(7):1950–64. doi: 10.1002/jad.70015 (PMC12493003; doi:10.1002/jad.70015)
Supplement: Supplementary file 1 — Agency in integrated pilot supplement. [file JAD-97-1950-s001.docx]

# SUPPLEMENT

Table 1: Integrated SRH & Economic Empowerment Intervention Elements by Geography

| Geography | **Ethiopia**  *Smart Steps* | **Northern Nigeria**  *MMA+* | **Southern Nigeria**  *9ja Girls+* |
| --- | --- | --- | --- |
| Target Audience | | | |
| Sub-Geography | Regions: Oromia;  Sidama; Southern Nations, Nationalities, and People | State: Kaduna | State: Ogun |
| Marital Status | Married | Married | Primarily unmarried |
| Target Age | 15-19 | 15-19 | 15-19 |
| Location | Rural | Peri-urban / Rural | Peri-urban / Urban |
| School Status | Out-of-school | Out-of-school / In-school | Out-of-school / In-school |
| Program Dosage | | | |
| Group Size | 10 - 15 | 24 | 20 |
| Meeting Frequency | Weekly | 2x weekly | 2x weekly |
| Session Duration | 1-2 hours | 90 minutes | 90 minutes |
| Program Duration | 30+ weeks | 12 weeks | 12 weeks |
| Program Elements | | | |
| Vocational Training |  | ● | ● |
| Business Training | ● | ● | ● |
| Financial Education | ● | ● | ● |
| Savings | ● | ● | ● |
| Asset Transfer | ● |  |  |
| Access to Low-Interest Loans | ● |  |  |
| Life Skills | ● | ● | ● |
| Critical Consciousness Building | ● | ● | ● |
| Mentorship / Coaching | ● | ● | ● |
| Group-based Learning / Peer Support | ● | ● | ● |
| Influencer Outreach | ● | ● | ● |
| SRH Knowledge (contraception) | ● | ● | ● |
| SRH Service Delivery (contraception) | ● | ● | ● |

Table 2: TIDIER Table for Intervention Description (Ethiopia)

| **Item Number** | **Item** |
| --- | --- |
| 1 | **Brief Name**  Integrated SRH and economic empowerment intervention (Smart Steps) |
| 2 | **Why**  The community of practice (governments, donors, implementers, and other local stakeholders) has shown increasing interest in addressing adolescent girls’ holistic needs through programming that combines sexual and reproductive health (SRH) with age-appropriate economic empowerment. A growing body of evidence demonstrates the types of interventions which demonstrate meaningful impact on adolescent girls’ economic empowerment. These include, for example, group-based models which are mentor-led and combine vocational training and business and soft skills development. Successful interventions also include the introduction of concepts related to gender and power to shift girls’ critical consciousness and are inclusive of follow-up after initial training, such as through mentorship or coaching. This integrated program design sought to integrate some of these evidence-based economic empowerment components into a well-established adolescent SRH intervention with the aim of improving girls’ economic and SRH outcomes as well as their agency. |
| 3 | **What**  Materials: Delivery of the SRH intervention components ([Smart Start](https://a360learninghub.org/the-interventions/ethiopia/)) relies primarily on a physical counseling tool ([the Smart Start counseling guide](https://a360learninghub.org/resource/smart-start-implementation-assets-counseling-guide/)) and also utilizes Ethiopian Ministry of Health (MOH) protocols and tools that govern the delivery of SRH services through the public sector across the country. Externally facing protocols and tools are available via the [Ethiopian MOH website](https://www.moh.gov.et/index.php/en/initiatives-4-col/Adolescent_and_Youth_Health_Program). The economic empowerment components utilizes: 1) [a flip book](https://a360learninghub.org/resource/ethiopia-smart-steps-asset-goal-setting-flip-book/) used to facilitate goal setting sessions, 2) [a member book](https://a360learninghub.org/resource/ethiopia-smart-steps-asset-member-book/) where girls record their individual savings and loan activity, 3) [a ledger book](https://a360learninghub.org/resource/ethiopia-smart-steps-asset-ledger-book/) which contains record-keeping instructions, the group’s accounting, and the group charter, 4) [a curriculum](https://a360learninghub.org/resource/ethiopia-smart-steps-asset-curriculum-guide/) used during group sessions to build girls’ soft and business skills. |
| 4 | **What**  Procedures: The integrated intervention begins with the SRH intervention components. Girls are mobilized from the community to attend an SRH counseling session with a Health Extension Worker (HEW) at a nearby health post. The HEW uses the Smart Start counseling guide to support girls to identify their goals and to understand how contraception can support them to achieve those goals. The HEW uses client-led counseling to recommend contraceptive methods based on a girl’s preferences and offers to provide the method. Interested girls are referred to a community-based program mentor who provides her with up to two goal setting sessions, the first with her husband and the second by herself. In these sessions, girls identify short and long-term goals and the steps to achieve them. Girls who participate are brought together in a group to form an empowerment collective (adapted from the Village Saving and Loan (VSLA) model). Groups start by establishing a charter, electing group leaders, and adopting rules for governing joint savings and loan behaviors. Girls meet once per week and contribute individual savings to a group savings pool. At each session the program mentor coaches the participants to oversee the savings and loan activities and periodically provides lessons to build girls’ soft and business skills. After 4 weeks of saving, girls can receive a loan from the group savings pool to invest in an income-generating activity which they are required to pay back within a period of 3 months. There are 18 lessons distributed across a period of approximately 10 months after which time girls can continue to contribute to groups savings and take loans from the group. Program mentors also provide individual support to help girls access further training or advance in their income generating activities. After 16 weeks groups were eligible for a matching grant of 150% of their total savings to increase the group’s loan pool if they met minimum standards for attendance, savings, and internal bookkeeping. After approximately seven months, the facilitator phased out and associations were fully self-governed. |
| 5 | **Who Provided**  The integrated intervention is delivered by three key categories of providers.  1) **Program Mentors:** These individuals are staffed in the community and are responsible for facilitation of the economic empowerment components of the integrated intervention along with some mobilization activities. Generally, the qualifications of program mentors include a minimum of 12^th^ grade completion, proficiency in reading and writing, fluency in local language, and prior experience working with youth in the community. Program mentors were mostly between the ages of 22 and 35. Program mentors were provided with training on program implementation strategy and objectives, participatory facilitation techniques, and youth-friendly program strategies.  2) **HEWs:** The Ethiopian health system relies on HEWS who deliver a package of essential services at the community level nation-wide. HEWs are provided pre-service and in-service training by the Ethiopian MOH. In the case of this integrated intervention, the SRH service delivery elements of the program are provided by HEWs.  3) **Womens Development Union (WDU) Members:** The WDU, previously known as the Womens Development Army (WDA) is a large unpaid community health workforce in Ethiopia intended to support improvements in population health. These community volunteers are selected based on their high status in the community and their healthy lifestyles and support in outreach and mobilization for health services. They receive minimal training by the Ethiopian MOH. In the case of this integrated intervention, WDU members support the mobilization of married girls for participation. |
| 6 | **How**  All intervention activities were provided through in-person engagement with participants. Mobilization of participants is done individually through home visits or with groups of girls in public spaces. SRH counseling is provided by a HEW to an individual girl or a couple. Goal setting sessions are provided by a program mentor to an individual girl and/or a couple. Girls receive support for pooling savings, taking loans, and developing soft skills in groups with the support of a program mentor. |
| 7 | **Where**  Mobilization for the intervention can be done through home visits or in community spaces. SRH counseling is conducted at the health facility or through home visits. Goal setting sessions is conducted in a public space or through home visits. Finally, all group-based program activities are conducted in community meeting spaces that are accessible to participants, most commonly at the nearest health facility. |
| 8 | **When and How Much**  The integrated intervention was delivered one time to a cohort of 400 married adolescent girls across 16 woredas in Oromia, Sidama, South, and Central regions in Ethiopia between June 2022 and April 2023. During this time participants each received a minimum of one SRH counseling session, up to two goal setting sessions with a program mentor, and the opportunity to attend up to 51 savings and loan session facilitated by a program mentor. The mentor also led 18 lessons that were combined with weekly group meetings. |
| 9 | **Tailoring**  N/A |
| 10 | **Modifications**  An additional component was added to reinforce program goals to participant’s husbands. In some communities they were resistant to their wife’s participation and additional outreach was needed to ensure smooth continuation of the savings groups. |
| 11 | **How Well: Planned**  Assessment of fidelity to implementation of the integrated intervention was planned through monitoring activities conducted by the program’s local implementing partners. Specific data gathered to assess fidelity included number of participants reached, number of groups formed, number of curriculum sessions facilitated and attended by participants, member contributions to group-based savings, and receipt / repayment of loans from the group savings pools by members. |
| 12 | **How Well: Actual**  Monitoring and evaluation activities demonstrated that over the pilot period 32 savings groups were formed (this included two per woreda, which was the intended target of the pilot). In terms of participant engagement, 99% of girls participated in at least one goal setting session, over 99% participated in a savings group, and over 70% did not miss any savings group sessions. Girls demonstrated consistent savings of around $1 per week per participant and over the pilot period over 700 loans were disbursed (meaning that many girls received and repaid more than one loan). |

Table 3: TIDIER Table for Intervention Description (Kaduna / Ogun)

| **Item Number** | **Item** |
| --- | --- |
| 1 | **Brief Name**  Integrated SRH and economic empowerment intervention (MMA+ / 9ja Girls +) |
| 2 | **Why**  The community of practice (governments, donors, implementers, and other local stakeholders) has shown increasing interest in addressing adolescent girls’ holistic needs through programming that combines sexual and reproductive health (SRH) with age-appropriate economic empowerment. A growing body of evidence demonstrates the types of interventions which demonstrate meaningful impact on adolescent girls’ economic empowerment. These include, for example, group-based models which are mentor-led and combine vocational training and business and soft skills development. Successful interventions also include the introduction of concepts related to gender and power to shift girls’ critical consciousness and are inclusive of follow-up after initial training, such as through mentorship or coaching. This integrated program design sought to integrate some of these evidence-based economic empowerment components into a well-established adolescent SRH intervention with the aim of improving girls’ economic and SRH outcomes as well as their agency. |
| 3 | **What**  The delivery of the SRH intervention components utilize Nigerian MOH counseling tools and protocols. These are not currently publicly available. The economic empowerment components are facilitated using a curriculum guide (one for the northern Nigeria intervention, [MMA+](https://a360learninghub.org/resource/mma-implementation-asset-curriculum-guide/), and one for the southern Nigeria intervention, [9ja Girls+](https://a360learninghub.org/resource/9ja-girls-implementation-tool-curriculum-guide/)). These curriculum sessions also utilize participant workbooks (for [MMA+](https://a360learninghub.org/resource/mma-implementation-asset-participant-workbook/) and [9ja Girls+](https://a360learninghub.org/resource/9ja-girls-implementation-asset-participant-workbook/) respectively). |
| 4 | **Procedures**  In southern and northern Nigeria, participants were mobilized to participate in the integrated intervention through program mentors who conducted home visits or mobilized girls in public spaces. In northern Nigeria girls were also referred to participate by their husbands who were engaged by male interpersonal communication agents (IPCAs) to understand the program objectives. In both southern and northern Nigeria, after mobilization participating girls attended five upskilling group sessions (90 minutes each) facilitated by program mentors. The first three sessions (called the ‘primary package’) focused on supporting girls to set goals for the future and build soft skills. In the last two sessions (called the ‘secondary package’) girls learned business skills, including how to manage their money. These sessions were conducted in the local public health centers (PHCs) and the option to access SRH service delivery on site was provided at each session. After completing both the primary and secondary curriculum package, girls elected to learn up to two vocational skills (over four to five weeks) through an apprenticeship (southern Nigeria) or vocational training center (northern Nigeria). Girls chose a variety of trades including catering, hairdressing, shoemaking, and photography. Concurrently, mentors offered support in developing and executing a business plan through individual and group-based counseling sessions. The program culminated in a large, public graduation that doubled as a marketplace for adolescent girls to display products and services. |
| 5 | **Who Provided**  The following individuals were responsible for delivering different aspects of the intervention:  1) Program mentors: These individuals facilitate the primary package for the integrated intervention. These are young women, below 40 years of age who can read and write and are recommended by community leaders or other individuals who are respected in the community. They receive training on the facilitators’ guide (specific content and objectives) and on participatory facilitation techniques.  2) Business mentors: These individuals facilitate the secondary package for the integrated intervention. They are experienced entrepreneurs with 5 or more years of experience and must be competent or certified in a minimum or two vocational skills. They must have a minimum of a completed secondary education, and a diploma or institutional degree is an advantage. In this case, they are expected to be female. They receive training on the expectations for delivering the curriculum content and on participatory facilitation techniques.  2) Health providers: The integrated intervention uses health providers staffed through the public health system which has its own requirements / criteria in their recruitment. In addition to the standard training provided by the Nigerian MOH to these providers, we provide additional training on adolescent-friendly and client-led SRH counseling techniques.  3) Interpersonal communication agents (IPCAs): For the key influencer engagement aspects of the intervention, IPCAs are used. They are expected to be male and the only major qualifications are their willingness to volunteer and their ability to read and write (either in English or Hausa). |
| 6 | **How**  All intervention activities were provided through in-person engagement with participants. Mobilization of participants is done individually through home visits or with groups of girls (or men) in public spaces. All skills curriculum sessions were delivered in-person to groups of girls. Mentorship sessions could be conducted either in groups or one-on-one with participants. |
| 7 | **Where**  Mobilization for the intervention was done in homes or in community spaces. Curriculum sessions were delivered at the health facility. Mentorship sessions could happen at the health facility, girls’ homes, or other community spaces convenient to participants. The health service delivery which happened concurrently with curriculum sessions took place at the health facility. |
| 8 | **When and How Much**  The integrated intervention was implemented with 2,927 girls across two local government authorities (LGAs) (Zaria and Sabon Gari) in Kaduna state and 953 girls across one LGA (Ado Odo Ota) in Ogun state over the period June 2022 to April 2023. Each participant received the opportunity to attend one goal setting session, three skill building sessions in the primary package, two skill building sessions in the secondary package, and additional vocational skills sessions or an apprenticeship over a period of four to five weeks. Participants could also access individual or group-based mentorship sessions with program mentors and limit was not imposed on the number of mentorship sessions participants could access. |
| 9 | **Tailoring**  N/A |
| 10 | **Modifications**  N/A |
| 11 | **How Well: Planned**  Assessment of fidelity to implementation of the integrated intervention was planned through monitoring activities conducted by the program’s local implementing partners and data collection for the pilot’s evaluation activities. Specific data gathered to assess fidelity included participation in core components of the intervention (goal setting, curriculum sessions, vocational skills, and mentorship sessions). |
| 12 | **How Well: Actual**  Data collection after the conclusion of the pilot demonstrated that over 98% of participants had engaged in a goal setting session. 91% and 86% of participants completed all the primary package sessions in Kaduna and Ogun respectively. 92% and 91% of participants completed all secondary package sessions in Kaduna and Ogun respectively. 98% of participants engaged in vocational skills training in both geographies. 99% of participants engaged in at least one mentorship session with 64% and 23% of participants engaging in more than two mentorship sessions in Kaduna and Ogun respectively. |

Table 4: Differential attrition analysis, Kaduna

| **Parameter** | **Category** | **Total n** | **Loss to follow up** | | | | **Sig.** |
| --- | --- | --- | --- | --- | --- | --- | --- |
|  |  |  | **Yes** | | **No** | |  |
|  |  |  | **n** | **%** | **n** | **%** |  |
| Program exposure | Comparison group | 493 | 126 | 25.6% | 367 | 74.4% | **<0.0001** |
|  | Intervention group | 556 | 82 | 14.7% | 474 | 85.3% |  |
| Purchased an asset | No | 270 | 51 | 18.9% | 219 | 81.1% | 0.7059 |
|  | Yes | 777 | 155 | 19.9% | 622 | 80.1% |  |
| Save money | No | 84 | 20 | 23.8% | 64 | 76.2% | 0.2872 |
|  | Yes | 244 | 45 | 18.4% | 199 | 81.6% |  |
| Earn money | No | 451 | 91 | 20.2% | 360 | 79.8% | 0.8055 |
|  | Yes | 598 | 117 | 19.6% | 481 | 80.4% |  |
| Earnings in the last 4 Weeks | No | 326 | 58 | 17.8% | 268 | 82.2% | 0.2665 |
|  | Yes | 723 | 150 | 20.7% | 573 | 79.3% |  |
| Earning in the last 7 Days | No | 370 | 68 | 18.4% | 302 | 81.6% | 0.3846 |
|  | Yes | 679 | 140 | 20.6% | 539 | 79.4% |  |
| Contribution to household expenses | No | 186 | 34 | 18.3% | 152 | 81.7% | 0.5944 |
|  | Yes | 412 | 83 | 20.1% | 329 | 79.9% |  |
| Currently using contraceptive | No | 640 | 127 | 19.8% | 513 | 80.2% | 0.9836 |
|  | Yes | 387 | 77 | 19.9% | 310 | 80.1% |  |
| Intend to use contraceptive in future | No | 177 | 41 | 23.2% | 136 | 76.8% | 0.1725 |
|  | Yes | 447 | 82 | 18.3% | 365 | 81.7% |  |
| Agency scale categories | Low | 967 | 196 | 20.3% | 771 | 79.7% | 0.2807 |
|  | Average | 75 | 12 | 16.0% | 63 | 84.0% |  |
|  | High | 7 | 0 | 0.0% | 7 | 100.0% |  |
| General Self-esteem categories | Low | 1 | 0 | 0.0% | 1 | 100.0% | 0.7985 |
|  | Average | 19 | 3 | 15.8% | 16 | 84.2% |  |
|  | High | 1028 | 205 | 19.9% | 823 | 80.1% |  |
| Self-Efficacy categories | Low | 28 | 6 | 21.4% | 22 | 78.6% | 0.4048 |
|  | Average | 228 | 52 | 22.8% | 176 | 77.2% |  |
|  | High | 791 | 149 | 18.8% | 642 | 81.2% |  |
| Age in years | 15 | 29 | 8 | 27.6% | 21 | 72.4% | 0.8311 |
|  | 16 | 43 | 8 | 18.6% | 35 | 81.4% |  |
|  | 17 | 114 | 23 | 20.2% | 91 | 79.8% |  |
|  | 18 | 353 | 66 | 18.7% | 287 | 81.3% |  |
|  | 19 | 510 | 103 | 20.2% | 407 | 79.8% |  |
| Highest level of education | Never attended school | 6 | 0 | 0.0% | 6 | 100.0% | 0.617 |
|  | Islamiyyah | 85 | 20 | 23.5% | 65 | 76.5% |  |
|  | Primary school | 101 | 24 | 23.8% | 77 | 76.2% |  |
|  | Junior secondary school | 206 | 40 | 19.4% | 166 | 80.6% |  |
|  | Senior secondary school | 546 | 105 | 19.2% | 441 | 80.8% |  |
|  | Above secondary | 105 | 19 | 18.1% | 86 | 81.9% |  |
| Ethnic group | Yoruba | 4 | 2 | 50.0% | 2 | 50.0% | 0.5964 |
|  | Hausa | 914 | 179 | 19.6% | 735 | 80.4% |  |
|  | Fulani | 78 | 17 | 21.8% | 61 | 78.2% |  |
|  | Igbo | 1 | 0 | 0.0% | 1 | 100.0% |  |
|  | Others | 52 | 10 | 19.2% | 42 | 80.8% |  |
| Number of children | None | 188 | 25 | 13.3% | 163 | 86.7% | 0.0929 |
|  | One child | 456 | 94 | 20.6% | 362 | 79.4% |  |
|  | Two children | 253 | 55 | 21.7% | 198 | 78.3% |  |
|  | Three or more children | 152 | 34 | 22.4% | 118 | 77.6% |  |
| Self-efficacy | Mean±SD |  | 3.2±0.6 |  | 3.3±0.6 |  | **0.0109** |
| Agency | Mean±SD |  | 1.4±0.3 |  | 1.4±0.4 |  | 0.0707 |
| Self-esteem | Mean±SD |  | 3.7±0.4 |  | 3.8±0.4 |  | **0.0306** |

Table 5: Differential attrition analysis, Ogun

| **Parameter** | **Category** | **Total n** | **Loss to follow up** | | | | **Sig.** |
| --- | --- | --- | --- | --- | --- | --- | --- |
|  |  |  | **Yes** | | **No** | |  |
|  |  |  | **n** | **%** | **n** | **%** |  |
| Program exposure | Comparison group | 426 | 168 | 39.4% | 258 | 60.6% | **<0.0001** |
|  | Intervention group | 501 | 95 | 19.0% | 406 | 81.0% |  |
| Purchased an asset | No | 281 | 63 | 22.4% | 218 | 77.6% | **0.0085** |
|  | Yes | 644 | 199 | 30.9% | 445 | 69.1% |  |
| Save money | No | 18 | 10 | 55.6% | 8 | 44.4% | 0.4936 |
|  | Yes | 92 | 43 | 46.7% | 49 | 53.3% |  |
| Earn money | No | 690 | 169 | 24.5% | 521 | 75.5% | **<0.0001** |
|  | Yes | 237 | 94 | 39.7% | 143 | 60.3% |  |
| Earnings in the last 4 weeks | No | 109 | 44 | 40.4% | 65 | 59.6% | **0.0031** |
|  | Yes | 818 | 219 | 26.8% | 599 | 73.2% |  |
| Earning in the last 7 days | No | 145 | 52 | 35.9% | 93 | 64.1% | **0.0294** |
|  | Yes | 782 | 211 | 27.0% | 571 | 73.0% |  |
| Contribution to household expenses | No | 134 | 51 | 38.1% | 83 | 61.9% | 0.5651 |
|  | Yes | 103 | 43 | 41.7% | 60 | 58.3% |  |
| Currently using contraceptive | No | 83 | 32 | 38.6% | 51 | 61.4% | 0.6685 |
|  | Yes | 245 | 101 | 41.2% | 144 | 58.8% |  |
| Intend to use contraceptive in future | No | 435 | 93 | 21.4% | 342 | 78.6% | 0.793 |
|  | Yes | 157 | 32 | 20.4% | 125 | 79.6% |  |
| Agency scale categories | Low | 520 | 124 | 23.8% | 396 | 76.2% | **0.0017** |
|  | Average | 298 | 98 | 32.9% | 200 | 67.1% |  |
|  | High | 109 | 41 | 37.6% | 68 | 62.4% |  |
| General Self-esteem Categories | Low | 6 | 1 | 16.7% | 5 | 83.3% | 0.5758 |
|  | Average | 100 | 32 | 32.0% | 68 | 68.0% |  |
|  | High | 821 | 230 | 28.0% | 591 | 72.0% |  |
| Self-efficacy category | Low | 14 | 3 | 21.4% | 11 | 78.6% | 0.0632 |
|  | Average | 207 | 72 | 34.8% | 135 | 65.2% |  |
|  | High | 705 | 188 | 26.7% | 517 | 73.3% |  |
| Age in years | 15 | 195 | 34 | 17.4% | 161 | 82.6% | **0.0002** |
|  | 16 | 138 | 31 | 22.5% | 107 | 77.5% |  |
|  | 17 | 154 | 46 | 29.9% | 108 | 70.1% |  |
|  | 18 | 188 | 64 | 34.0% | 124 | 66.0% |  |
|  | 19 | 252 | 88 | 34.9% | 164 | 65.1% |  |
| Highest level of education | Never attended school | 8 | 3 | 37.5% | 5 | 62.5% | 0.4506 |
|  | Islamiyyah | 1 | 1 | 100.0% | 0 | 0.0% |  |
|  | Primary school | 23 | 8 | 34.8% | 15 | 65.2% |  |
|  | Junior secondary school | 190 | 47 | 24.7% | 143 | 75.3% |  |
|  | Senior secondary school | 654 | 190 | 29.1% | 464 | 70.9% |  |
|  | Above secondary | 51 | 14 | 27.5% | 37 | 72.5% |  |
| Ethnic group | Yoruba | 865 | 249 | 28.8% | 616 | 71.2% | 0.3944 |
|  | Igbo | 29 | 5 | 17.2% | 24 | 82.8% |  |
|  | Others | 33 | 9 | 27.3% | 24 | 72.7% |  |
| Number of Children | None | 770 | 205 | 26.6% | 565 | 73.4% | **0.0471** |
|  | One child | 104 | 41 | 39.4% | 63 | 60.6% |  |
|  | Two children | 29 | 10 | 34.5% | 19 | 65.5% |  |
|  | Three or more children | 24 | 7 | 29.2% | 17 | 70.8% |  |
| Married/staying as Married |  | 851 | 234 | 27.5% | 617 | 72.5% | **0.0482** |
|  |  | 76 | 29 | 38.2% | 47 | 61.8% |  |
| Self-efficacy | Mean±SD |  | 3.2±0.5 |  | 3.3±0.6 |  | 0.5712 |
| Agency | Mean±SD |  | 2.0±0.8 |  | 1.7±0.8 |  | **0.0001** |
| Self esteem | Mean±SD |  | 3.4±0.5 |  | 3.5±0.5 |  | 0.3351 |

Table 6: Unadjusted and adjusted difference-in-difference (DiD) for agency-related outcomes in economic empowerment pilot evaluation, revised using propensity score weights

| Panel A: Unadjusted DiD | | | | | | | | | | | | | | | | | |  |
| --- | --- | --- | --- | --- | --- | --- | --- | --- | --- | --- | --- | --- | --- | --- | --- | --- | --- | --- |
|  | **Decision-making Power** | | | | | | **Mobility** | | | | | | **Self-Efficacy** | | | | | |
|  | Ethiopia | | Kaduna | | Ogun | | Ethiopia | | Kaduna | | Ogun | | Ethiopia | | Kaduna | | Ogun | |
|  | *n=1600* | | *n=1890* | | *n=1591* | | *n=1600* | | *n=1890* | | *n=1591* | | *n=1600* | | *n=1890* | | *n=1591* | |
|  | **Baseline** | **Endline** | **Baseline** | **Endline** | **Baseline** | **Endline** | **Baseline** | **Endline** | **Baseline** | **Endline** | **Baseline** | **Endline** | **Baseline** | **Endline** | **Baseline** | **Endline** | **Baseline** | **Endline** |
|  | *M(SD)* | *M(SD)* | *M(SD)* | *M(SD)* | *M(SD)* | *M(SD)* | *M(SD)* | *M(SD)* | *M(SD)* | *M(SD)* | *M(SD)* | *M(SD)* | *M(SD)* | *M(SD)* | *M(SD)* | *M(SD)* | *M(SD)* | *M(SD)* |
| Intervention | 2.04 (0.48) | 2.15 (0.36) | 1.61 (0.55) | 2.33 (0.67) | 2.44 (0.45) | 2.86 (0.25) | 2.03 (0.88) | 2.21 (0.82) | 1.48 (0.43) | 1.55 (0.37) | 1.57 (0.66) | 2.41 (0.66) | 3.34 (0.53) | 3.52 (0.43) | 3.40 (0.50) | 3.78 (0.31) | 3.22 (0.56) | 3.56 (0.43) |
| Control | 2.08 (0.43) | 2.10 (0.37) | 2.10 (0.68) | 2.52 (0.59) | 2.55 (0.43) | 2.45 (0.40) | 2.11 (0.82) | 2.28 (0.78) | 1.32 (0.29) | 1.48 (0.36) | 2.10 (0.80) | 2.14(0.69) | 3.52 (0.43) | 3.34 (0.53) | 3.16 (0.63) | 3.59 (0.38) | 3.31 (0.55) | 2.93 (0.53) |
| **Unadjusted DiD** | 0.08 | | **0.32***** | | **0.59***** | | 0.003 | | **-0.08*** | | **0.90***** | | **0.36***** | | -0.03 | | **0.75***** | |
|  | (-0.002; 0.16) | | **(0.23; 0.42)** | | **(0.50; 0.68)** | | (-0.16; 0.17) | | **(-0.14; -0.02)** | | **(0.75; 1.05)** | | **(0.27; 0.45)** | | (-0.11; 0.06) | | **(0.64; 0.87)** | |
|  |  |  |  |  |  |  |  |  |  |  |  |  |  |  |  |  |  |  |
| Panel B: Adjusted DiD | | | | | | | | | | | | | | | | | | |
|  | **Decision-making Power** | | | | | | **Mobility** | | | | | | **Self-Efficacy** | | | | | |
|  | Ethiopia | | Kaduna | | Ogun | | Ethiopia | | Kaduna | | Ogun | | Ethiopia | | Kaduna | | Ogun | |
|  | *n=1600* | | *n=1890* | | *n=1591* | | *n=1600* | | *n=1890* | | *n=1591* | | *n=1600* | | *n=1890* | | *n=1591* | |
| **Time*Intervention (DiD)** | 0.06(-0.02; 0.14) | | **0.34(0.24; 0.43)***** | | **0.61(0.52; 0.70)***** | | -0.02(-0.19; 0.14) | | **-0.08(-0.14; -0.01)*** | | **0.96(0.81; 1.11)***** | | **0.35(0.25; 0.44)***** | | -0.03(-0.12; 0.06) | | **0.76(0.65; 0.88)***** | |
| **Program Exposure** |  |  |  |  |  |  |  |  |  |  |  |  |  |  |  |  |  |  |
| Comparison | Ref. | | Ref. | | Ref. | | Ref. | | Ref. | | Ref. | | Ref. | | Ref. | | Ref. | |
| Intervention | -0.02(-0.08; 0.04) | | -0.65(-0.73; -0.56)*** | | -0.17(-0.24; -0.10)*** | | -0.05(-0.17; 0.06) | | 0.13(0.09; 0.18)*** | | -0.65(-0.76; -0.53)*** | | -0.18(-0.25; -0.11)*** | | 0.10(0.03; 0.18)** | | -0.13(-0.22; -0.04)** | |
| **Period** |  |  |  |  |  |  |  |  |  |  |  |  |  |  |  |  |  |  |
| Baseline | Ref. | | Ref. | | Ref. | | Ref. | | Ref. | | Ref. | | Ref. | | Ref. | | Ref. | |
| Endline | 0.01(-0.05; 0.06) | | 0.39(0.32; 0.46)*** | | -0.18(-0.25; -0.10)*** | | 0.15(0.04; 0.26)** | | 0.13(0.09; 0.18)*** | | -0.09(-0.22; 0.04) | | -0.18(-0.24; -0.11)*** | | 0.42(0.34; 0.49)*** | | -0.43(-0.53; -0.33)*** | |
| **Age** |  |  |  |  |  |  |  |  |  |  |  |  |  |  |  |  |  |  |
| Age 15-17 | Ref. | | Ref. | | Ref. | | Ref. | | Ref. | | Ref. | | Ref. | | Ref. | | Ref. | |
| Age 18-19 | 0.19(0.14; 0.23)*** | | 0.13(0.03; 0.22)** | | 0.08(0.04; 0.12)*** | | 0.30(0.21; 0.39)*** | | 0.04(-0.01; 0.09) | | 0.11(0.03; 0.19)** | | 0.01(-0.05; 0.07) | | 0.09(0.01; 0.16)* | | 0.02(-0.04; 0.07) | |
| **Married/ Living as Married** |  |  |  |  |  |  |  |  |  |  |  |  |  |  |  |  |  |  |
| No | N/A | | N/A | | Ref. | | N/A | | N/A | | Ref. | | N/A | | N/A | | Ref. | |
| Yes | N/A | | N/A | | -0.22(-0.32; -0.12)*** | | N/A | | N/A | | -0.21(-0.39; -0.03)* | | N/A | | N/A | | -0.03(-0.16; 0.09) | |
| **Highest Level of Education** |  |  |  |  |  |  |  |  |  |  |  |  |  |  |  |  |  |  |
| Primary and Below | Ref. | | Ref. | | Ref. | | Ref. | | Ref. | | Ref. | | Ref. | | Ref. | | Ref. | |
| Secondary | 0.02(-0.03; 0.06) | | 0.30(0.21; 0.39)*** | | 0.11(-0.04; 0.26) | 0.11(-0.04; 0.26) | 0.03(-0.06; 0.13) | | 0.04(-0.01; 0.08) | 0.04(-0.01; 0.08) | 0.29(0.09; 0.48)** | 0.29(0.09; 0.48)** | 0.01(-0.04; 0.07) | | 0.28(0.21; 0.35)*** | 0.28(0.21; 0.35)*** | 0.11(-0.06; 0.28) | 0.11(-0.06; 0.28) |
| Above Secondary | 0.02(-0.15; 0.19) | | 0.37(0.25; 0.50)*** | | 0.16(-0.02; 0.33) | 0.16(-0.02; 0.33) | 0.18(-0.17; 0.53) | | 0.08(0.01; 0.15)* | 0.08(0.01; 0.15)* | 0.48(0.21; 0.74)*** | 0.48(0.21; 0.74)*** | -0.03(-0.24; 0.17) | | 0.32(0.24; 0.41)*** | 0.32(0.24; 0.41)*** | 0.25(0.04; 0.45)* | 0.25(0.04; 0.45)* |
| **Parity** |  |  |  |  |  |  |  |  |  |  |  |  |  |  |  |  |  |  |
| 0 | Ref. | | Ref. | | Ref. | | Ref. | | Ref. | | Ref. | | Ref. | | Ref. | | Ref. | |
| 1 | 0.04(-0.01; 0.09) | | -0.16(-0.24; -0.07)*** | | 0.08(0.00; 0.15)* | | 0.09(-0.01; 0.19) | | -0.01(-0.07; 0.05) | | 0.36(0.21; 0.51)*** | | 0.04(-0.02; 0.10) | | -0.05(-0.13; 0.03) | | 0.16(0.06; 0.25)*** | |
| ≥2 | 0.06(0.00; 0.12)* | | -0.15(-0.24; -0.06)*** | | 0.13(0.07; 0.19)*** | | 0.20(0.08; 0.32)* | | 0.03(-0.04; 0.09) | | 0.35(0.24; 0.46)*** | | 0.01(-0.06; 0.08) | | -0.07(-0.15; 0.00) | | 0.06(0.00; 0.13) | |

*Table 7: Categorization of decision-making power and mobility / permission scales*

| Category | Range | Categorization |
| --- | --- | --- |
| Decision-making Power | | |
| Dependent Decision-making | 1-1.49 | Has responded ‘someone else does’ to more than half of the prompts |
| Joint Decision-making | 1.5-2.49 | Has responded ‘I do with someone else’ to at least two prompts |
| Independent Decision-making | 2.5-3.0 | Has responded ‘I do’ to at least two prompts |
| Mobility | | |
| Limited Mobility | 1-2.24 | Responses are mostly ‘always’ or ‘sometimes’ |
| Occasional Mobility | 2.25-3.24 | Responses are mostly ‘sometimes’ or ‘rarely’ |
| Frequent Mobility | 3.25-4 | Responses are mostly ‘rarely’ or ‘never’ |

Table 8: Categorical analysis of agency outcomes by geography and group

|  | Ethiopia | | | | Kaduna | | | | Ogun | | | |
| --- | --- | --- | --- | --- | --- | --- | --- | --- | --- | --- | --- | --- |
| **Overall** | **Baseline** | | **Endline** | | **Baseline** | | **Endline** | | **Baseline** | | **Endline** | |
|  | n | % | n | % | n | % | n | % | n | % | n | % |
| Very High | 80 | 10% | 84 | 11% | 3 | 0% | 1 | 0% | 60 | 9% | 155 | 23% |
| High | 117 | 15% | 141 | 18% | 22 | 3% | 72 | 9% | 148 | 22% | 258 | 39% |
| Medium | 257 | 32% | 328 | 41% | 212 | 25% | 433 | 52% | 217 | 33% | 193 | 29% |
| Low | 302 | 38% | 244 | 31% | 363 | 43% | 253 | 30% | 225 | 34% | 54 | 8% |
| Very Low | 44 | 6% | 3 | 0.4% | 241 | 29% | 82 | 10% | 14 | 2% | 4 | 0.6% |
| **Comparison** | **Baseline** | | **Endline** | | **Baseline** | | **Endline** | | **Baseline** | | **Endline** | |
| Very High | 36 | 9% | 39 | 10% | 0 | 0% | 1 | 0% | 50 | 19% | 26 | 10% |
| High | 56 | 14% | 75 | 19% | 7 | 2% | 36 | 10% | 76 | 30% | 92 | 36% |
| Medium | 136 | 34% | 177 | 44% | 148 | 40% | 210 | 57% | 63 | 24% | 90 | 35% |
| Low | 156 | 39% | 107 | 27% | 141 | 38% | 92 | 25% | 65 | 25% | 47 | 18% |
| Very Low | 16 | 4% | 2 | 1% | 71 | 19% | 28 | 8% | 4 | 2% | 3 | 1% |
| **Intervention** | **Baseline** | | **Endline** | | **Baseline** | | **Endline** | | **Baseline** | | **Endline** | |
| Very High | 44 | 11% | 45 | 11% | 3 | 1% | 0 | 0% | 10 | 3% | 129 | 32% |
| High | 61 | 15% | 66 | 17% | 15 | 3% | 36 | 8% | 72 | 18% | 166 | 41% |
| Medium | 121 | 30% | 151 | 38% | 64 | 14% | 223 | 47% | 154 | 38% | 103 | 25% |
| Low | 146 | 37% | 137 | 34% | 222 | 47% | 161 | 34% | 160 | 39% | 7 | 2% |
| Very Low | 28 | 7% | 1 | 0% | 170 | 36% | 54 | 11% | 10 | 3% | 1 | 0.3% |
|  | χ^2^ (4, 800) = 5.49, p =.240 | | χ^2^ (4, 800) = 7.09, p =.131 | | χ^2^ (4, 841) = 85.71, p<.001 | | χ^2^ (4, 841) = 15.08, p=.005 | | χ^2^ (4, 664) = 78.53, p<.001 | | χ^2^ (4, 664) = 92.80, p<.001 | |
