# Supplementary material for: Adolescent Girls' Agency in an Integrated Sexual and Reproductive Health and Economic Empowerment Intervention Pilot
Source: J Adolesc. 2025 Jul 8;97(7):1950–64. doi: 10.1002/jad.70015 (PMC12493003; doi:10.1002/jad.70015)
Supplement: Supplementary file 2 — Supplement Figure 1 Composite agency categories. [file JAD-97-1950-s002.pdf]

	Limited Mobility	Occasional Mobility	Frequent Mobility
**Dependent Decision-making**	1 – Very Low	2 – Low	3 – Medium
**Joint Decision-making**	2 – Low	3 – Medium	4 – High
**Independent Decision-making**	3 – Medium	4 – High	5 – Very High
